# General functions to transform associate data to host data, and their use in phylogenetic inference from sequences with intra-individual variability

**DOI:** 10.1186/1471-2148-8-86

**Published:** 2008-03-18

**Authors:** Markus Göker, Guido W Grimm

**Affiliations:** 1 Organismic Botany, Eberhard-Karls-University, Auf der Morgenstelle 1, Tübingen, Germany; 2 Institute of Geosciences, Eberhard-Karls-University, Sigwartstrasse 10, Tübingen, Germany

## Abstract

**Background:**

Amongst the most commonly used molecular markers for plant phylogenetic studies are the nuclear ribosomal internal transcribed spacers (ITS). Intra-individual variability of these multicopy regions is a very common phenomenon in plants, the causes of which are debated in literature. Phylogenetic reconstruction under these conditions is inherently difficult. Our approach is to consider this problem as a special case of the general biological question of how to infer the characteristics of hosts (represented here by plant individuals) from features of their associates (represented by cloned sequences here).

**Results:**

Six general transformation functions are introduced, covering the transformation of associate characters to discrete and continuous host characters, and the transformation of associate distances to host distances. A pure distance-based framework is established in which these transformation functions are applied to ITS sequences collected from the angiosperm genera *Acer*, *Fagus *and *Zelkova*. The formulae are also applied to allelic data of three different loci obtained from *Rosa *spp. The functions are validated by (1) phylogeny-independent measures of treelikeness; (2) correlation with independent host characters; (3) visualization using splits graphs and comparison with published data on the test organisms. The results agree well with these three measures and the datasets examined as well as with the theoretical predictions and previous results in the literature. High-quality distance matrices are obtained with four of the six transformation formulae. We demonstrate that one of them represents a generalization of the Sørensen coefficient, which is widely applied in ecology.

**Conclusion:**

Because of their generality, the transformation functions may be applied to a wide range of biological problems that are interpretable in terms of hosts and associates. Regarding cloned sequences, the formulae have a high potential to accurately reflect evolutionary relationships within angiosperm genera, and to identify hybrids and ancestral taxa. These results corroborate earlier ones which showed that treelikeness measures are a valuable tool in comparative studies of biological distance functions.

## Background

Multicopy gene regions such as the nuclear ribosomal DNA spacers may exhibit significant heterogeneity within the same individual. Such intra-individual variability can cause serious problems for phylogenetic reconstructions that use a dichotomous tree as the general model of evolution, and has been generally referred to as "paralogy" [1-6]. The internal transcribed spacers ITS1 and ITS2 of the nuclear DNA region encoding for the 18S-5.8S-25S ribosomal RNA cistron, the 35S rDNA are amongst the most commonly used molecular markers for plant phylogenetic studies [3,5]. As far as it has been studied, intra-individual ITS variability is a very common phenomenon in plants [7-24] and has been attributed to numerous causes, such as (i) incomplete concerted evolution among the multiple copies of 35S rDNA located within the same nucleolus organizer region (NOR); (ii) the general potential of the NOR regions for intragenomic recombination between both parental chromosomes; (iii) ITS homoeology, i.e. the persistence of two or more independently inherited arrays of 35S rDNA as found for allopolyploids with more than one NOR; (iv) ITS pseudogeny, i.e. the occurrence of non-functional copies of rRNA genes, and (v) gene paralogy in a strict sense, i.e. the existence of several rDNA loci coding for *functionally differing *rRNAs. Whereas incomplete concerted evolution, intragenomic recombination, ITS homoeology and ITS pseudogenes have been documented in [[1,9,12,14,16,18,22,23] and [25]] (among many others) and can be considered to be natural phenomena, paralogs have not yet been observed in the case of the 35S rDNA, but have been observed in the case of "oocyte-type" and "somatic" 5S rRNA genes active during early development of *Xenopus laevis *[26-29].

Cloned sequence data that effectively cover the intra- and inter-individual ITS variability of morphologically defined taxa have been subject to detailed studies in angiosperm genera such as *Acer *(Sapindales, Sapindaceae) [9,11,30], *Fagus *(Fagales, Fagaceae) [9,23,24,30] and *Zelkova *(Rosales, Ulmaceae) [22]. Significantly high ITS variability not linked to pseudogeny was found particularly in species that are considered to be diploid; hence, other effects in addition to ITS homoeology were taken into account for the observed heterogeneity. For *Acer *and *Fagus *species, it could be shown that intra-individual ITS variability contained strong taxonomic information: Morphologically distinguishable but closely related taxa ("species" or "subspecies") exhibited identical or highly similar (< 1% sequence divergence) ITS variants in addition to taxon-specific ITS variants not found in the sister taxon [9,11,23,24,30]. In particular, intra-individual ITS variability in *Fagus *was found to be as high as or higher than the overall interspecific divergence [23,24]. This puzzling observation fitted well with the fossil history of this genus, which indicates several phases of unhindered horizontal gene flow among spatially and temporarily isolated "species" during the Tertiary, as well as known ecological differences between the species [23]. In an experimental approach, Grimm et al. [31] coded the occurrence or lack of a certain nucleotide polymorphism as phylogenetic characters for species-based matrices, which were analysed with maximum parsimony and maximum likelihood. A nucleotide matrix comprising several cloned ITS sequences per individual and a number of individuals per morphologically defined "species" was transformed into a matrix of characters of the "STANDARD" type of the NEXUS format used by PAUP* [32], with the morphologically defined taxa and biogeographically circumscribed "populations" as Operational Taxonomic Units (OTUs). Sites reflecting no polymorphism (e.g., always "C") were coded as "0", whereas their polymorphic counterparts (e.g., "C" and "T") were coded as "1". More complex variability patterns were coded using up to eight character states. As a result, Grimm et al. [31] inferred a more detailed molecular phylogenetic hypothesis than would have been possible by using the primary (untransformed) sequence data, supporting the phylogenetic scenario predicted earlier [23].

Despite those promising results, the coding of the occurrence or loss of a nucleotide polymorphism as a phylogenetic character of a predefined species can be methodologically critical. The mutational constraints affecting intra- and inter-genomic competition among ITS variants are not as well studied as the degree of intra-genomic recombination. Knowing this could allow us to decide if it makes sense to regard the occurrence (or lack of) a nucleotide polymorphism as a character under maximum parsimony, and would be crucial to define the likelihood that such a polymorphism occurs quantitatively. To minimize coding bias, Grimm *et al*. [9] relied on detailed visual investigations of the nucleotide sequence motifs and general polymorphic patterns (further details given in [30]), a procedure which was extremely time-consuming and, to some extent, subjective. One could avoid such problems if one could develop a function that allows us to convert a data matrix of the clones into a data matrix of the plant individuals (or of greater taxonomic units) directly.

However, due to the multiple reasons for ITS polymorphism discussed in the literature (above), it is, at present, hard to rely on an explicit statistical model of the evolution of multiple copies to infer the phylogeny of the individuals. For instance, Joly and Bruneau [33] described a distance transformation method to include allelic variation in phylogenetic reconstruction, which can only be applied if exactly one or exactly two alleles are present in each individual. In contrast, our approach is based on the notion that different biological phenomena can be described in terms of *hosts *and their *associates*, and that similar methodologies can be applied to solve questions related to the different types of host-associate pairs. Host-associate pairs are, for instance, areas and species, host species and their parasite or mutualist species, or individual organisms and individual genes [34-36]. Methods such as Brooks Parsimony Analysis (BPA) have been suggested as a means of inferring area cladograms from species trees as well as host trees from parasite or mutualist trees [37-39]. In the following, we thus derive and empirically test approaches that could be applied directly (or after minor modifications) to all kind of associate character data, be it morphological or sequence data of individuals in certain geographic areas or habitats, parasites or mutualists on certain host organisms, or genes and sequence variants present in certain individuals. The obvious advantage is that any method found to be useful for cloned ITS sequences and plant individuals may also be useful in other fields of research dealing with host-associate pairs [34-36].

In contrast to the original formulation of BPA [37], which infers host *trees *from single to multiple associate *trees*, we here will confine ourselves to distance methods in combination with phylogenetic networks for several reasons. Firstly, both trees and networks can be inferred from distance matrices, but networks represent many evolutionary processes in a more comprehensive manner than trees, and evolution is not necessarily treelike [40-44]. For similar reasons, Joly and Bruneau [33] also relied on networks in examining their distance transformation method for allelic data. Whereas distance methods have already been criticized for losing phylogenetic information more than two decades ago [45], they may even represent the data better than common character-based methods, particularly in combination with network analyses [44], which could not yet well be combined with the maximum-likelihood criterion. Secondly, the phylogenetic quality (treelikeness) of distance matrices may be measured directly without deriving a tree. Instead, helpful but apparently underused indices such as Q values [46], Delta values [47-49], or additivity (as implemented in OVW; [50]) can be applied. Thirdly, congruence between datasets can be tested directly on distance matrices [51,52]. While concordance between trees is mostly measured with formulae, such as the Robinson-Foulds distance [53], which disregard branch length information and do not indicate whether the similarity between the trees is significant (e.g., [54]: p. 528–535), permutational regression approaches for distance matrices [51,52] allow one to test the null hypothesis of no congruence between the datasets. Even though permutational regression could be applied to patristic (path-length) distances derived from trees with branch lengths [55], it may be more appropriate to use the "noisier" pair-wise phenetic distances (e.g., [56]), particularly if they are not well represented by a tree [40-44]. This is also more efficient, since inferring trees (as two additional computational steps) can be omitted.

**Figure 1:**
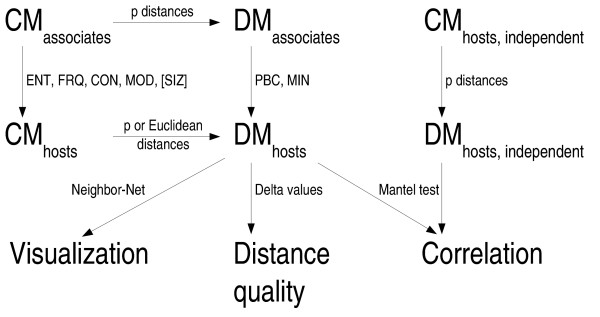
**Overview of the distance methods and the tests applied**. The picture describes the pure-distance framework developed in the course of the present study for testing methods of deriving host character data and/or distance matrices from associate character data. The location of the methods for character-character transformation (ENT, FRQ, CON, MOD) and for distance-distance (MIN, PBC) transformation is indicated. Integrating the SIZ distances (that are biologically meaningless in the case of cloned sequences) allows us to test for a potential sampling size bias. Abbreviations: CM, character matrix; DM, distance matrix. See the methods section for the abbreviations for the transformation methods.

In principle, derivation of host from associate distance matrices can be done by two means (Fig. 1). Either (i) the associates' character matrix is converted to a character matrix of the hosts, the latter being used to compute host distances, or (ii) distances between the associates are computed from the associates' character matrix and the resultant distance matrix is converted to a distance matrix of the hosts. An example of the latter approach, restricted to up to two associates per host, is presented in the study of Joly and Bruneau [33].

In the present study, we apply such functions to compute distances between plant individuals as operational taxonomic units (as the hosts) from molecular data matrices that exhibit significant intra-taxon sequence heterogeneity reflected by cloned ITS data (as the associates). The quality (treelikeness) of distance matrices is estimated using the Delta value approach [47-49]. Delta values can also be calculated for individual taxa and may be indicative of hybridization and/or recombination [49]. To identify the distance formulae best suited for the tasks of interest, we also investigate the correlation between the resultant distance matrices and matrices based on morphological distances as an external, independent dataset; this investigation will include permutational statistical tests [52]. Whereas morphological data are often regarded as intrinsically inferior to molecular characters, more thorough analyses do not confirm that view [57]. In the context of a parsimony framework, congruence between (molecular and/or morphological) datasets has been suggested as a selection criterion for character change costs in sequence alignment and a subsequent tree search [58]; here, we use congruence to compare associate-host transformation methods. To be able to correlate morphology and cloned ITS data covering the intra-individual variability of corresponding individuals, we focus on the above-mentioned datasets of *Acer *section *Acer *[9], *Fagus *[23,59] and *Zelkova *[22]. For these taxa, literature data indicate that the respective patterns of morphological and ITS differentiation fit into uniform evolutionary and systematic concepts, i.e., are qualitatively congruent (see above). Thus, correlation of morphological and transformed molecular matrices represents a natural means of quantitatively validating the proposed transformations.

We also test the sensitivity of each distance function against sample numbers of associates per hosts (here, the number of clones that represent an individual), making use of the above-mentioned permutational test, and against gap handling techniques (as "missing data" or as a "5^th ^state" over and above the four nucleotides). Finally, the outcome of the analyses is discussed briefly in the light of the evolutionary framework for each group, as established in the original literature, using a combination of ITS data, morphology and fossil evidence. As additional empirical tests, we apply the transformation functions to the three datasets presented in [33]; the resulting networks are compared to those obtained with the transformation formula presented in Joly and Bruneau [33].

In addition to this empirical assessment of the distance formulae, implementations of these algorithms are provided as executables for the three most popular operating systems. Regarding Delta Values, we also provide a comprehensive implementation that significantly extends the capabilities of the Python script used in [48], which is, to the best of our knowledge, the only implementation available so far.

## Results

### Sampling size bias, distance quality and correlation with morphology

Correlations of the transformed distances with SIZ distances, which were designed for detecting a potential bias related to sampling size (i.e., the number of cloned sequences obtained per plant individual), were statistically significant in some cases (SIZ distance matrices are provided in Additional file 1; full correlation matrices, including Spearman correlation values and permutational probabilities as calculated with CADM, are provided in Additional file 2). In *Acer*, a significant correlation between PBC and SIZ distances was observed if a sampling size threshold of 2 was applied and gaps were treated as missing character states; the correlation disappeared if the minimum number of associates required was incremented by 1 (and therefore, we omitted plant individuals for which fewer than three sequences were obtained). Whereas molecular distances inferred from *Fagus *sequences never displayed significant correlations with SIZ, the correlation of *Zelkova *SIZ and ENT distances as obtained by treating gaps as missing character states and by applying a threshold of 2 was significant, but this also became insignificant if the minimum number of associates required was increased. Therefore, in the following, we confined ourselves to the results obtained with a threshold of 3, which resulted in host datasets comprising 39, 28 and 11 taxa for *Acer*, *Fagus *and *Zelkova*, respectively.

**Figure 2:**
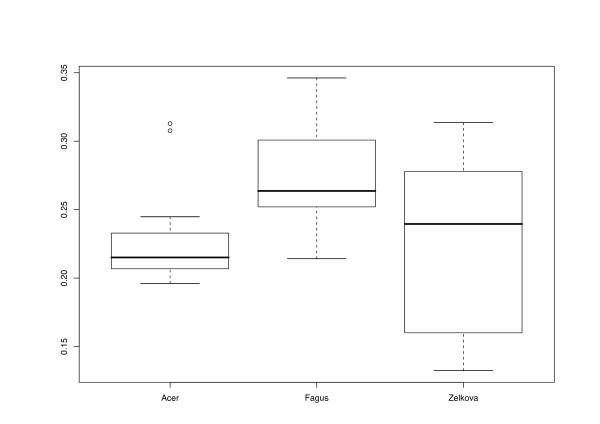
**Data source vs. Delta values**. Delta values (computed with DIST_STATS) of distance matrices obtained with a minimum of three associates plotted against data sources, i.e. the plant genera from which the cloned ITS sequences were obtained. Lower Delta values indicate higher treelikeness of the distance matrices. The boxplots indicate the positions of medians (thick horizontal lines), quartiles (boxes), outliers (short horizontal lines connected to the box with dashed lines), and extreme values (open circles).

Regarding Delta values (DV) for entire distance matrices, results differed between data sources (Fig. 2; all DV are provided in Additional file 1, along with all the distance matrices obtained). *Acer *distances showed relatively low values, indicating high treelikeness; variability between the different methods applied was low. The DV for *Acer *distances were mostly located between 0.189 and 0.245, and received higher values with ENT distances only. In *Fagus*, the DV were much higher in general (0.214–0.346) and displayed more differences between the distance methods applied. In *Zelkova*, the overall highest treelikeness was observed (a DV of 0.132 was obtained with MOD distances and gaps treated as a 5^th ^character state), but variability between the distance functions was also highest (up to a DV of 0.314 for ENT distances combined, with gaps treated as missing data).

**Figure 3:**
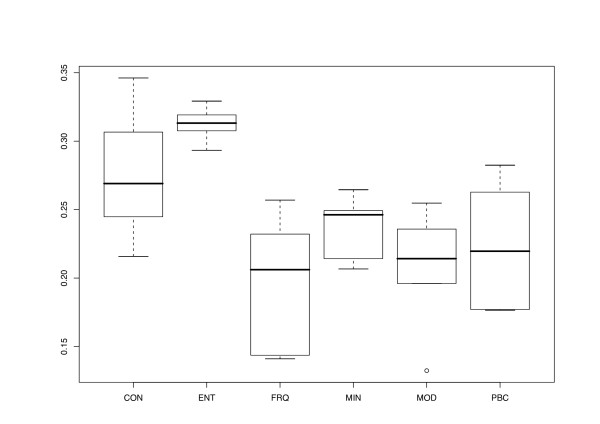
**Transformation method vs. Delta values**. Delta values plotted against transformation method, that is, the character-character or distance-distance transformation the final distances relied on. For further explanations, see legend to Fig. 2.

**Figure 4:**
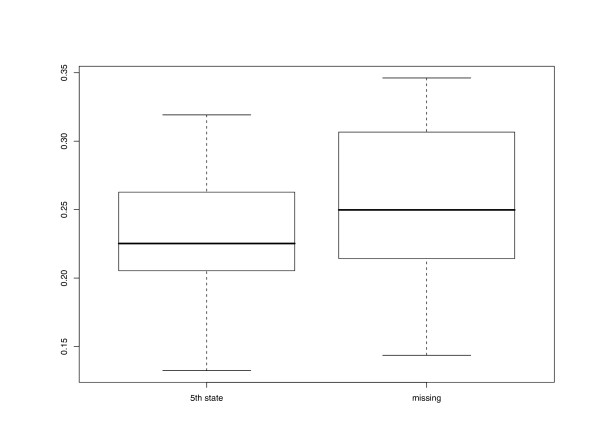
**Gap treatment vs. Delta values**. Delta values plotted against the gap treatment applied in CON or MOD character-character transformations and in computation of p distances. For further explanations, see legend to Fig. 2.

The different character and distance transformation functions resulted in even more pronounced differences regarding the treelikeness of the matrices (Fig. 3). ENT distances rather uniformly produced high DV (0.293–0.329). Treelikeness of CON matrices was even lower in some cases, whereas variability was much higher (0.216 – 0.346). MIN and PBC DV were generally much lower and similar in size to each other, but variability was more pronounced in the latter. MOD and FRQ achieved the lowest DV and were also similar in the variance of their treelikeness. In general, DV were lower if distance matrices were inferred and gaps were treated as a 5^th^character state (Fig. 4).

**Figure 5:**
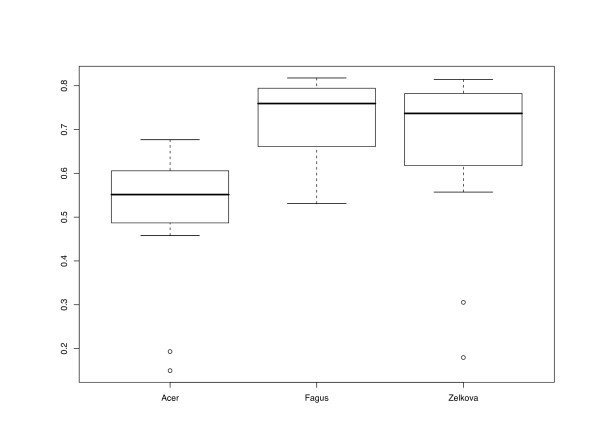
**Data source vs. correlation with morphology**. Spearman correlation coefficients (computed with CADM) between molecular distance matrices obtained with a minimum of three associates and morphological distance matrices plotted against plant genera from which the cloned ITS sequences were obtained. For further explanations, see legend to Fig. 2.

The correlation between molecular and morphological distances was significant at p = 0.01 in all cases except for all *Acer *ENT distances as well as *Zelkova *ENT distances combined with gaps treated as a missing character state; these distances correspond to the correlation coefficients below 0.3 in Fig. 5. In contrast to the results for treelikeness, on average, correlation values are highest for *Fagus *and lowest for *Acer*. MIN/5^th ^state for Fagus (0.818), FRQ/missing for *Zelkova *(0.814) and PBC/missing for *Acer *(0.677) received the best correlation with the corresponding morphological matrices.

**Figure 6:**
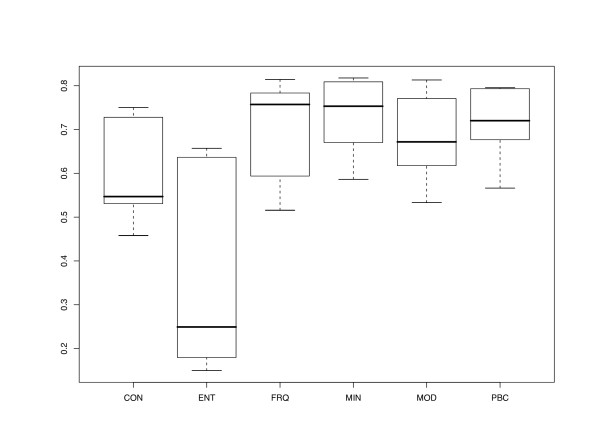
**Transformation method vs. correlation with morphology**. Correlation coefficients plotted against transformation method, i.e. the character-character or distance-distance transformation the final distances relied on. For further explanations, see legend to Figs. 2 and 5.

**Figure 7:**
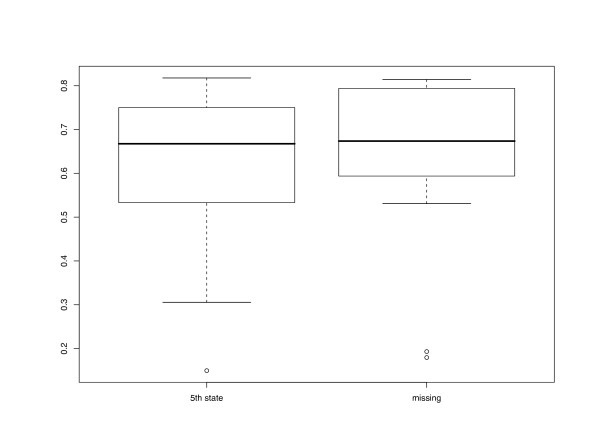
**Gap treatment vs. correlation with morphology**. Correlation coefficients plotted against the gap treatment applied when computing p distances. For further explanations, see legends to Figs. 2 and 5.

Regarding the differences between the transformation functions, the behaviour of the correlation coefficients (Fig. 6) paralleled that of the DV: formulae that resulted in lower DV also displayed higher correlations with the morphological distances. The correlation was lowest in the case of ENT (even though the variance is considerable), followed by CON. FRQ, MIN, MOD and PBC displayed relatively high correlation values, ranging from 0.516 (*Acer*/FRQ/5^th ^state) to 0.818 (*Fagus*/MIN/5^th ^state). On average, correlation coefficients were higher if gaps were treated as missing character states (Fig. 7), even though the corresponding DV were lower.

**Figure 8:**
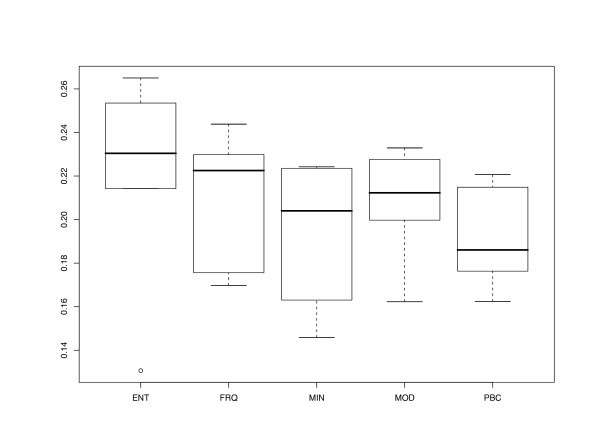
**Transformation method vs. Delta values for *Rosa *data**. Delta values plotted against transformation method as in Fig. 2, but for glyceraldehyde 3-phosphate dehydrogenase, triose phosphate isomerase and malate synthase alleles obtained from North American *Rosa *individuals [33]. The CON method was not depicted because results for up to two associates per host are identical to those obtained with MOD.

Results obtained for North American *Rosa *sequences [33] (the complete results are shown in Additional file 3) were similar to those described above. With a single exception, the DV were considerably larger for ENT than for the other transformation functions (Fig. 8); MOD distance matrices (equivalent to CON for up to two associates per host) were somewhat less treelike than those of FRQ, MIN and PBC. In the case of the *Rosa *datasets, treating gaps as a fifth state resulted in a lower DV (not shown). Triose phosphate isomerase sequences resulted in less treelike matrices than glyceraldehyde 3-phosphate dehydrogenase and malate synthase sequences (not shown).

### Individual Delta values

The individual DV (iDV) based on the best performing distance transformations (FRQ, MIN, MOD and PBC combined with gaps treated as missing data) varied considerably between plant specimens (Table 1 and Additional file 4). Markedly increased iDV in *Acer *section *Acer *were found in representatives of group B4 (*A. saccharum *subspecies; accessions **gd 1**, **ni A**) and individual **us 11 **(*A*. cf. *monspessulanum*; iDV > 0.28 with PBC, FRQ, MOD; Table 1). The pattern was most pronounced in **ni A **(highest iDV) and **us 11 **(2^nd ^highest iDV). Individuals of potentially hybrid origin with homoeologous ITS sequences showed medium to comparably low iDV, e.g. *A*. × *pseudo-heldreichii *individual **hd 1**: 0.168 (FRQ) to 0.197 (PBC), **xx 5 **(*A. monspessulanum *with partly recombinant ITS): 0.169 (MOD) to 0.260 (MIN). In *Fagus*, iDV were generally high (e.g., 0.224 to 0.379 for PBC). Above average iDV were characteristic of individuals of *F. hayatae*, *F. longipetiolata*, *F. lucida *and *F. sylvatica *from Turkey and Georgia, whereas below average iDV were typically found in *F. grandifolia *and subgenus *Engleriana *(*F. japonica*, *F. engleriana*). *Zelkova *had the lowest iDV (e.g., between 0.144 and 0.237 for PBC). Here, the MIN and MOD transformations produced less treelike data (iDV > 0.20/0.19 respectively) than FRQ and PBC; based on PBC, the only iDV exceeding 0.2 was confined to individual **cp 2**. The same individual also showed the maximum iDV with FRQ, where all iDV were below 0.2. The hybrid individual **se 2 **(*Z*. cf. *serrata*, based on ITS: *Z. schneideriana *× *Z. serrata*) had low iDV with PBC and FRQ, and high or even maximal iDV with MIN and MOD.

**Table 1:** Individual Delta values (iDV) of the plant individuals.

	PBC	FRQ	MIN	MOD	Evolutionary interpretation
*Acer *section *Acer*, median iDV	0.217	0.200	0.215	0.214	
Individual **gd 1**, group B4, *A. saccharum*	0.279	0.268	0.255	0.299	Early isolated lineage; maximal iDV (**gd **with a strong plesiomorphic signal)
Population **ni A**, group B4, *A. saccharum*	0.315	0.305	0.328	0.319	
Individual **us11**, group B2, *A*. cf. *monspessulanum*	0.294	0.310	0.283	0.300	Ancestral within group B2
Individual **ib 1**, group B2, *A. ibericum*	0.230	0.198	0.224	0.256	2^nd ^highest iDV in group B2
Individual **ms17**, group B2, *A. monspessulanum*	0.188	0.182	0.161	0.168	Minimal iDV, highly diagnostic ITS sequences
Individual **xx 5**, group B2, *A. monspessulanum*	0.230	0.198	0.260	0.169	Includes two chimeric clones (B2 × B3)
*Fagus*, median iDV	0.276	0.255	0.248	0.248	
Individual **lo47**, subgenus *Fagus*: *F. longipetiolata*	0.379	0.352	0.335	0.337	Maximal iDV, reflecting an ancient ITS polymorphism
Individual **lo 2**, subgenus *Fagus*: *F. longipetiolata*	0.279	0.250	0.272	0.256	Lowest iDV in *F. hayatae-longipetiolata*
Individual **ja25**, subgenus *Engleriana*: *F. japonica*	0.224	0.194	0.215	0.193	Minimal iDV; all ITS variants of subgenus *Engleriana *are clearly distinct from ot her *Fagus *spp.
*Zelkova*, median iDV	0.172	0.142	0.246	0.228	
Individual **cp 2**, *Z. carpinifolia*	0.237	0.182	0.246	0.265	Ancestral within genus; highest iDV
Individual **se 2**, *Z*. cf. *serrata *(hybrid)	0.160	0.136	0.252	0.300	Genetic hybrid of *Z. serrata *and *Z. schneideriana*
Individual **se 5**, *Z. serrata*	0.167	0.142	0.266	0.228	
Individual **sd 1**, *Z. schneideriana*	0.178	0.155	0.240	0.203	
Individual **sd 3**, *Z. schneideriana*	0.144	0.112	0.201	0.219	Minimal iDV, only species-diagnostic ITS variants

Plant specimens' iDV inferred from distances matrices obtained with four different transformation methods are compared; gaps were treated as missing data and a minimum number of three associates per host was applied. Affiliation to plant species as well as an evolutionary interpretation of particularly high iDV (i.e. low treelikeness) observed is indicated.

The iDV of the plant individuals mostly reflected the iDV calculated directly from p distances between the cloned sequences (treating gaps as missing data; also included in Additional file 4). For *Acer*, all clones of the *A. saccharum *subspecies (group B4) exhibited the largest iDV (0.207–0.333), as did the clones of the individual **us 11 **(0.252 – 0.273). The same is true for chimeric (recombinant) clones (e.g., **xx 3**, **xx 5**, **sv4b**). In *Fagus*, a similar correlation between iDV of clones and plant individuals was found; for instance, the minimal iDV of 0.238 was obtained for clones of **gr-5104 **(individual **gr 51**, *F. grandifolia *from Mexico) in contrast to 0.387 for the clone **or-618 **(individual **or 6**, *F. sylvatica *from northwest Turkey). For *Zelkova*, a comparison of iDV of clones with iDV of plant individuals revealed that FRQ and PBC reflect a major feature of the original character or distance data: Four of the six clones representing **cp 2 **were among the 18 clones with iDV > 0.250, but only one clone of either **si 2 **(highest iDV based on MIN) and **se 2 **(MOD) were in this group.

### Phylogenetic networks

**Figure 9:**
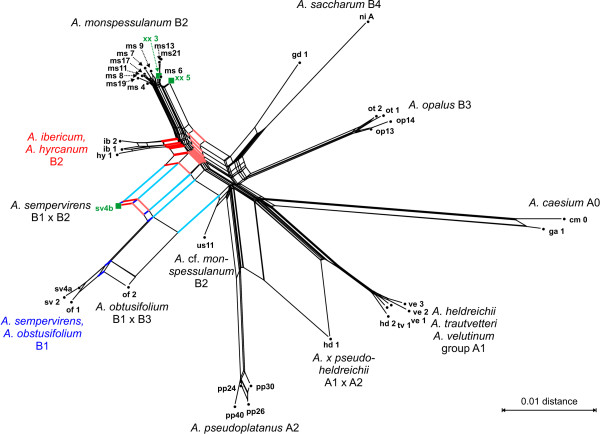
**Neighbor-Net splits graph based on PBC-inferred distances: *Acer *section *Acer***. This graph largely agrees with the results of [9]: Individuals (bold labels) of the same taxon ('species') and intrasectional group (A0 to B4) cluster together. The position of potentially hybrid individuals (**hd 1**, **of 2**, **sv4b**) is pronounced and is directly visible from the graph because of the large proximal box-like portions. These hybrid individuals exhibit ITS clones of different evolutionary origin (ITS homoeologues), highlighted for the example of individual **sv4b**. Clones of this individual either represented *A. ibericum*-type or group B1-type homoeologues. Related edge bundles (reddish, bluish) can be addressed in the graph. Green squares correspond to individuals that exhibited recombinant (chimeric) clones (see text).

Naturally, the similarity between the reconstructed networks correlates with the congruence between the underlying distance matrices. The four transformation methods (FRQ, MIN, MOD and PBC) that resulted in high correlations with morphology and in low DV produced very similar Neighbor-Net splits graphs (details not shown, but Additional file 1 contains all distance matrices calculated for import in SplitsTree, as well as the pair-wise correlations between all distance matrices). In the following, we thus focus on the description of the networks based on PBC distances. The PBC-inferred network of *Acer *section *Acer *(Fig. 9) exhibited two general elements. First, genetically unambiguous individuals (or local groups of co-occurring, taxonomically identical individuals) clustered according to their taxonomic affiliations ("species" and "subspecies") and the intra-sectional groups A0 to B4 (Fig. 9). Each group, except for group B2, was characterized by prominent parallel edges and a rather treelike appearance in the corresponding portions of the graph. Second, three individuals were placed as terminals of large box-like structures: (1) **hd 1**, representing a putative hybrid of *A. heldreichii *and *A. pseudoplatanus *(*A*. × *pseudo-heldreichii*), (2) **of 2**, material from a historical herbarium sheet of *A. obtusifolium *from Syria, and (3) **sv4b**, a morphologically unequivocal *A. sempervirens *individual from Crete. All three individuals were characterized by potential ITS homoeologues [9]. Aside these two major features, the following could be observed: The individual **us 11**, taxonomically treated as *A*. cf. *monspessulanum*, was placed near the center of the graph; the most prominent edge bundles related it to group B1 or A2. The center of the graph was dominated by relatively short edges; however, the two most prominent central edges related group B1 to group B2, and group A1 to the (potential) outgroup A0.

**Figure 10:**
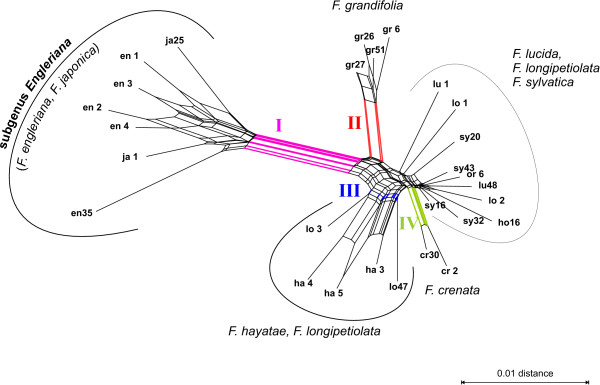
**Neighbor-Net splits graph based on PBC-inferred distances: *Fagus***. Individuals are grouped according to their general patterns of ITS variability [23, 31]. For example, generally high intra- and inter-individual variability that is diagnostic at a taxonomic level for subgenus *Engleriana*, but also *F. hayatae*-*F. longipetiolata*, is reflected by long edge bundles forming prominent proximal box-like structures. Coloured edges with Roman numerals refer to the text.

In the PBC network of *Fagus *(Fig. 10), four major groups could be distinguished. A long edge bundle distinguished between the two subgenera *Engleriana *(*F. engleriana*, *F. japonica*; **I**) and *Fagus *(all other species). Within the latter, individuals representing the Northern American *F. grandifolia *(**II**) were clearly separated from the remaining Eurasian taxa. Of the Eurasian taxa, individuals of *F. hayatae *and *F. longipetiolata *(**III**, except **lo 1 **and **lo 2**) were placed next to each other as terminals of a pronounced box-like substructure. All other individuals were organized in a star-like manner; the longer proximal edge bundles further related both individuals of *F. crenata *(**IV**).

**Figure 11:**
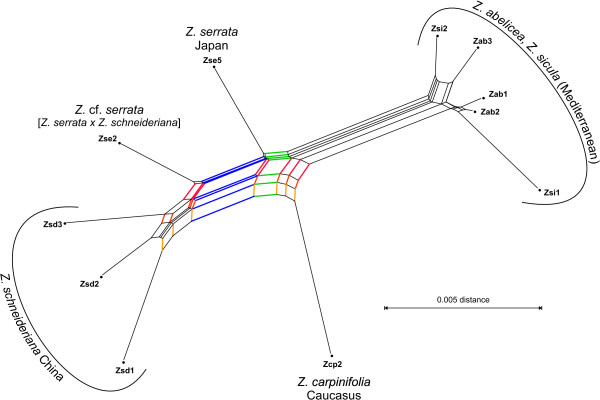
**Neighbor-Net splits graph based on PBC-inferred distances: *Zelkova***. A strong (palaeo-) biogeographic pattern is visible, which was established in the course of the evolutionary unfolding of this genus [22]. The placement of each species and individual correlates perfectly with its genetic (considering the potential hybrid individual **Zse 2**) and evolutionary background, as sketched in [22], and highlighted by coloured edge bundles. Further colours used are: • blue, edge bundle distinguishing between the Chinese *Z. schneideriana *(and **Zse 2 **exhibiting *Z. schneideriana*-type ITS homoeologues) and the closely related other species; • reddish, edge bundles reflecting the evolutionary pathway from *Z. carpinifolia *(primitive, shares edges with the sister taxon *Z. schneideriana*) through *Z. serrata *(more derived, secondary reticulation with *Z. schneideriana*) towards *Z. abelicae *and *Z. sicula *(most derived); • green, edge bundle that correlates with differential relationships between and among Eastern Asian and Western Eurasian extant species, addressed in detail in [22].

The PBC-inferred Neighbor-Net splits graph based on ITS data of *Zelkova *(Fig. 11) had a "giraffe-like" general appearance, reflecting a biogeographical differentiation pattern. A prominent box-like structure (the "torso" of the giraffe) was created by *Z. carpinifolia *from the Caucasus (individual **cp 2 **made the "foreleg"), individuals representing the Chinese *Z. schneideriana *that made the "hind legs" and "tail", and a French *Z*. cf. *serrata *cultivar (**se 2**) and the Japanese-based *Z. serrata *(**se 5**) that formed the backbone. The "head" of the splits graph giraffe was separated from the "torso" by a pronounced "neck" comprising the Mediterranean *Zelkova *individuals of the (very) closely related species *Z. abelicea *and *Z. sicula*.

**Figure 12:**
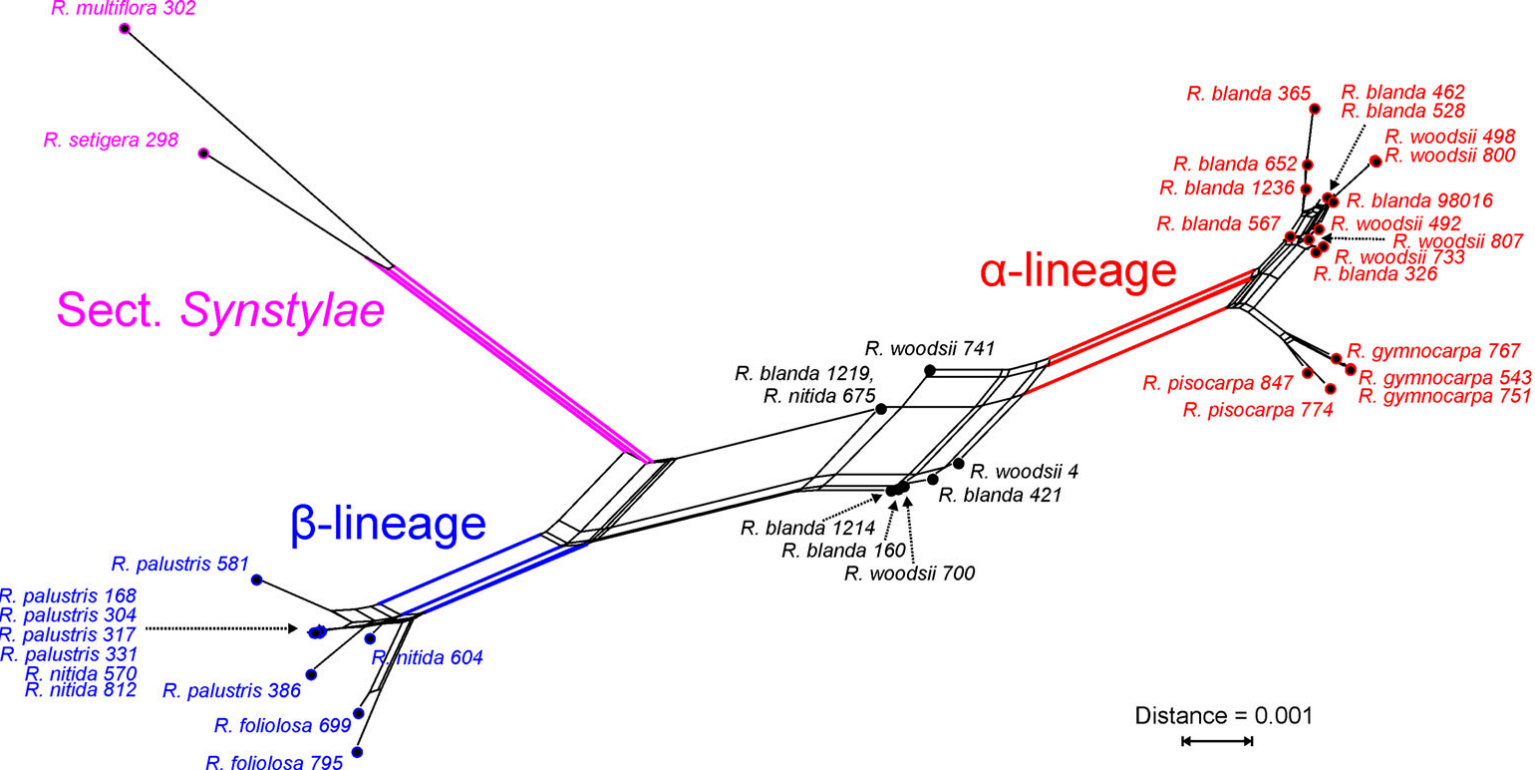
**Neighbor-Net splits graph based on PBC-inferred distances: *Rosa *malate synthase data**. The network topology is fully compatible with the splits graph depicted in [33], Fig. 5, which is based on the same sequence data and a different transformation algorithm. Colours refer to the three lineages indicated in Fig. 5 of [33], all of which are also obtained in our PBC network.

Regarding the Neighbor-Net splits graphs of the *Rosa *data, we chose to depict the PBC network computed from malate synthase sequences (Fig. 12) because an annotated network inferred from the same sequence alignment using a different transformation algorithm was shown in Fig. 5 of [33]. The resulting network topologies are fully compatible. Three lineages, alpha, beta and *Rosa *section *Synstylae*, were indicated in [33], all of which are recovered in the PBC network (Fig. 12). Topological relationships within and between these lineages as well as with the remaining OTUs are also congruent with those obtained by earlier works [33]. As in the case of *Acer*, *Fagus *and *Zelkova*, the correlation between FRQ, MIN, MOD and PBC distances were high (almost always > 0.9), and the resulting splits graphs were similar to each other and those obtained in [33] (not shown; splits graphs can be inferred from the distance matrices contained in Additional file 3, which also contains all correlation matrices).

## Discussion

### General applicability of the transformation formulae

Of the six character-character or distance-distance transformations examined in the course of the present study, four (FRQ, MOD, MIN and PBC) performed well with respect to both treelikeness of the resulting distance matrices and correlation with morphology. In the case of the *Rosa *datasets, the resulting network topologies are fully in agreement with those obtained by Joly and Bruneau [33], who used a distance transformation function restricted to up to two associates per host. In the following, we will focus on the technical applicability of the six transformation formulae; we will also show that while some formulae that are characterized by a combination of reasonable performance and apparent simplicity (such as FRQ and MIN), others are less simple, but can be justified by their relation to coefficients that are well-known from statistics (ENT, MOD and PBC). Before, we note that the considerable agreement observed between four of the six transformation functions and the morphological distances also confirms the use of latter as reference datasets. This is not circular reasoning; rather, because congruence between distinct data sources is a reliable indicator that they reflect the same underlying evolutionary history (e.g., [50]), the high quality of the morphological datasets [22,23,59] is a necessary condition for obtaining high correlation values.

Obviously, both ENT and FRQ can be applied to each type of discrete character data. Only in the case of quantitative and continuous characters such as morphological measurements need they be replaced by other appropriate character transformations. For instance, ENT could be replaced by the standard deviation or the range as typical parametric or non-parametric statistics for the variability of a sample. In fact, the statistical justification of the entropy formula (formula 1) requires some considerations from information theory but is as widely used as a coefficient for the variability of discrete data as are the aforementioned statistics for continuous data (e.g., [60]: p 209, 240). FRQ could be applied to quantitative, continuous characters after transforming them to discrete characters by means of partitioning them into several clusters. However, since most molecular (e.g. aligned nucleotide or amino acid sequences) and morphological characters (e.g. coded as presence/absence or as multiple discrete states) are represented as a set of discrete character states, ENT and FRQ can be applied directly in most cases.

The unfavourable performance of ENT distances with respect to both Delta values and correlation with morphology is no surprise. We presume that ENT distances will always almost discard at least some phylogenetic information and that they will usually discard more information than their counterparts such as FRQ. Thus, ENT distances may not represent the best method to uncover evolutionary relationships between hosts. However, they may be of use to clarify if and to what extent the common ancestry of the hosts is reflected by the variability of associate characters alone. For instance, the correlation between *Acer *and *Zelkova *ENT distances and the corresponding morphological distances was extremely low and mostly insignificant, whereas *Fagus *ENT distances significantly correlated with morphology (see Additional file 2). This corroborates earlier studies which concluded that the distribution and amount of intra-individual ITS variability in *Fagus *directly reflects phylogenetic relationships [23,30,31].

A further difference between ENT and FRQ is that ENT may be generally more susceptible to sample size, as demonstrated by the significant correlation with SIZ distances for our data. However, testing the correlation with SIZ represents an easy means to correct for a potential sample size bias, because raising the minimum required number of associates to 3 resulted in insignificant correlations of SIZ distances with all other formulae. Of course, if the number of associates has a biological meaning (as in case of the *Rosa *data), the correlation with SIZ cannot be used as a selection criterion. A drawback of ENT and FRQ is that they cannot be combined with distance formulae (or methods of phylogenetic inferences) that make use of an explicit model of DNA (or amino acid) evolution (e.g., [54,61]). However, both transformation methods could be combined with bootstrapping, the standard method in phylogeny to obtain branch support values (e.g., [54,61]), if an implementation is available to bootstrap continuous characters (currently, this cannot be done with, for example, PAUP* [32].) In the case of FRQ, block bootstrapping would need to be applied, treating each sequence of character state frequencies that was computed from the same character within the original data matrix as a single, independent character. In the current study, for the reasons provided in the background section, we have preferred networks over trees and bootstrapping, and we have omitted any of the more complex models of nucleotide site substitution, as explained in the methods section.

As in the case of ENT, the low performance of the CON transformation is not surprising. Because character states that only appear once receive the same weight than highly frequent states, a considerable loss of information may occur. If all possible character states are present in the set of associates of a single host, loss of information is total (resulting in "N" or "X" in the case of nucleotides). Naturally, this problem has been noted earlier [33]. In contrast to ENT, CON keeps track of the actual character states present and thus the results are better in general (Figs. 3, 6). However, CON cannot be used to assess the correspondence between associate character variability and host phylogeny (as can ENT), and because character transformations that perform better regarding phylogenetic reconstruction have been found, a biological justification for the use of the CON transformation of associate characters is not obvious. In fact, CON has been included in the current study mainly because it makes use of the standard approach to compute consensus sequences and to compare its performance with that of other formulae qualitatively and quantitatively.

As expected, in the case of a minimum number of associates (three), MOD considerably outperforms CON. Determining the mode is the standard method of obtaining a representative value from qualitative characters, as are the mean and median statistics for continuous data (e.g., [60]: p. 185; [62]: p. 48). Most probably, the loss of information is much lower than in the case of the CON transformation, except for hosts with exactly two associates [33]. Furthermore, with very high numbers of divergent associates, MOD will also result in "N" or "X" characters only (in case of nucleotides), whereas FRQ may tend to equal proportions of each character state. On the other hand, it is reasonable to assume that, in general, MOD loses more information than FRQ because MOD disregards all character states that occur with less than maximum frequency. In case of *Z. carpinifolia*, differences between the iDV of the cloned ITS sequences are less well preserved by MOD (and MIN) than by FRQ (and PBC), as further discussed below. This may be considered as preliminary evidence for the differences between the four transformation methods regarding the loss of information, even though all perform well with respect to overall treelikeness and correlation with independent morphological datasets. An advantage of MOD (and CON) is that the same methods of phylogenetic inference can be applied to transformed as well as untransformed characters. Thus, the full range of methods for phylogeny reconstruction, including Maximum Parsimony and Maximum Likelihood, are available, which could also be combined with bootstrapping (as implemented in, for example, PAUP* [32]) to obtain branch support values (e.g., [54,61]).

The general applicability of the PBC distance formula is demonstrated by its relationship to the Sørensen distance (or similarity) coefficient familiar to ecology (e.g., [60]: p. 256 and 275; [63]). Sørensen distances are frequently used to visualize relationships between habitats based on the presence and absence of species. An extension of Sørensen's coefficient introduced by Odum, as well as by Bray and Curtis, allows us to take species abundances into account [60]: p. 265 and 287. Sørensen's coefficient is known as an "asymmetric" distance coefficient, because double absence of a species from two habitats is not taken into account; rather, the Sørensen distance D_xy _between two habitats (hosts) x and y relates the number of species (associates) a_xy _present in both habitats to the number of species present in x only (a_x_) and present in y only (a_y_) in the following way (e.g., [60]: p. 256 and 275):

(4) *D*_*xy*_: = (*a*_*x *_+ *a*_*y*_)/(2a_*xy *_+ *a*_*x *_+ *a*_*y*_)

The denominator of the Sørensen distance (Formula 4) is always identical to that of the PBC distance (Formula 3), because PBC adds the number of rows to the number of columns of the reduced matrix (see the methods section). Thus, PBC counts associates that occur in both hosts twice, and those that occur in only one of the two hosts once, respectively. Importantly, the numerator of PBC (Formula 3) becomes identical to that of the Sørensen distances if PBC is computed from a phylogenetically uninformative associate distance matrix that contains 0 in the diagonal and 1 in all remaining fields. In that case, the sum of the row minima is identical to a_i _because 0 is added for each associate being identical and 1 is added for each associate being different in the two hosts; the sum of the column minima is identical to a_j _for the same reasons. Of course, if a phylogenetically uninformative distance matrix that contains any positive constant k other than 1 in the non-diagonal fields is used as input, the resulting distance will be identical to k times the Sørensen distance; this just represents a means of rescaling.

For these theoretical reasons, we conclude that PBC is a biologically meaningful generalization of Sørensen's distance coefficient to take the phylogenetic relationships between the associates into account. From the viewpoint of the more general PBC formula (Formula 3), Sørensen distances are a special case that is based on a distance matrix that contains information only on identity (zero distances) and non-identity (positive constant k as distances) but no further information on phylogenetic relationships. Accordingly, the new distance formula (Formula 3) has been christened "Phylogenetic Bray-Curtis" after another extension of Sørensen's coefficient by Bray and Curtis (e.g., [60], p. 265 and 287), abbreviated "PBC".

It is reasonable to assume that PBC does not suffer from the limitations of ENT, FRQ, CON and MOD, as described above. Even in the case of a large number of highly divergent associates, the most similar associates of the second host will be determined by the PBC algorithm. Importantly, there is a difference in perspective compared to the transformation function presented by Joly and Bruneau [33]. Even if the latter was extended to more than two associates per host, in the case of an equal number of associates, it would assume that each associate of the first host can only be linked to a single associate of the second host. If and only if this assumption is justified biologically, the algorithm of Joly and Bruneau [33] or an extension of it may be the preferred transformation. However, our more general algorithms appear to work well with the *Rosa *datasets, for which the assumption is reasonable.

Like PBC, MIN is also justified by statistical considerations. Minimum, maximum and median are standard non-parametric statistics (e.g., [60]: p. 185; [62]: p. 45), but only the minimum can be applied directly in transforming distances (see the methods section). MIN has an advantage over PBC because it is simpler. However, it may be hypothesized that MIN loses more information than PBC because the latter considers a larger proportion of values in the reduced distance matrix. As mentioned above, results obtained for the iDV of *Z. carpinifolia *are in agreement with that prediction. On the other hand, if the original dataset consisted of several clearly distinct associate lineages, PBC would be heavily influenced by the loss of all members of any of the associate lineages in one or more hosts because the row or column minima would increase considerably. This behaviour of PBC may be desirable with many datasets, such as several independent lineages of parasites on the same host lineage, as in the original BPA approach to infer the host from associate cladograms [37].

To solve other biological questions, MIN may be the more appropriate transformation. For similar reasons, PBC is more heavily influenced by sampling size than MIN, as indicated by the significant correlation with SIZ observed with a low threshold value. Both MIN and PBC could be integrated in an analysis pipeline that makes use of standard phylogenetic techniques such as an explicit evolutionary model and bootstrapping (see above). In contrast to CON and MOD, these methods would need to be applied before transformation, not afterwards. Joly and Bruneau [33] emphasized the combination of transformed datasets from several loci for phylogenetic analyses. MIN- or PBC-transformed distance matrices would require averaging [33], which can be done using a Perl script provided together with the transformation programs (see below). Integrating the transformed character obtained using CON, MOD, FRQ and ENT for the same sets of hosts is straightforward because the datasets just need to be concatenated and the number of characters updated.

### Phylogenetic implications

The splits graphs based on FRQ, MIN, MOD and PBC distances are in agreement with the evolutionary frameworks that have been proposed for *Acer *section *Acer *[9], *Fagus *[23] and *Zelkova *[22]. Because congruence between these distance matrices and morphology is well documented by the correlation results, for more details the reader is referred to the original literature. In the following, we will focus on the evolutionary interpretation of the iDV obtained from these distances and whether they are able to recover treelikeness observed within the matrices of cloned ITS sequences. As demonstrated below, networks based on the four character or distance transformations obviously have a high capability to deal simultaneously with a couple of "paralogy" phenomena that interfere with phylogenetic tree-building, such as "recombinant" accessions, ITS homoeologues and reticulate signal in general. These considerations also corroborate earlier results that DV are a valuable tool for comparative studies of biological distance functions [48].

### Individual Delta values and the recognition of ancestral taxa

Two taxa in *Acer *section *Acer *show generally high iDV, based on the original molecular distances between the cloned sequences as well as on the distance transformations. These are representatives of *A. saccharum *and its subspecies, and the *A*. cf. *monspessulanum *individual **us 11**. Grimm *et al*. [11] noted that *Acer *section *Acer *falls into two major lineages: *A. caesium *and the *Acer *core clade, which finds further support in cpDNA data [64]. Nevertheless, the ITS sequence structure hints towards *A. caesium *as the closest extant relative of the *Acer *core clade [11,30]. According to [9], *A. saccharum *(group B4) is at the tip of an isolated lineage that originated shortly after the formation of the *Acer *core clade (respective section *Acer*). Based on motif analysis, they showed that subspecies *grandidentatum *is closer to the common ancestor of the whole section than its eastern relatives and that it has retained several plesiomorphic sequence characters. An extant sister clade can not be identified. The high DV mirror this finding. Analogously, *A*. cf. *monspessulanum *shows a largely "primitive" ITS sequence, from which all ITS variants of group B2 can be derived [9]. In a cladistic analysis, an ancestor would be placed as sister taxon to all its offspring, thus producing a polytomy if more than one sibling is included in the dataset. In network analysis, several incompatible phylogenetic splits are promoted, reflecting a generally non-treelike mode of evolution in agreement with the high iDV.

The comparably high DV found for *Fagus *are in agreement with the observation that (molecular) evolution in this genus is non-treelike in general [23,30]. Accordingly, the ITS data accumulated a high amount of incompatible signals [24,31], which interfere with phylogenetic tree-building and result in high DV, but can be handled using adapted analyses, as done in [31] and the present study.

Morphology and ITS motif analyses [31] have provided evidence that *Z. carpinifolia *is a more primitive member of that genus, and could be a remainder of the evolutionary source (ancestral population) from which *Z. serrata *and *Z. abelicea-sicula *have evolved. In analogy to *A*. cf. *monspessulanum *and *A. saccharum *(subsp. *grandidentatum*), the iDV of *Z. carpinifolia*, based on the distances between the clones, on FRQ and on PBC distances, are higher than those of other individuals. This pattern is not apparent with MOD and MIN distances, which may be caused by greater loss of information in the case of the latter transformations (see above).

### Transformed distances and the recognition of hybrids and recombinants

Grimm *et al*. noted the existence of putative ITS homoeologues in a few individuals and documented "recombinant" (chimeric) clones in the case of *Acer *section *Acer *[9]. In analyses based on the original cloned ITS data, the putative ITS homoeologues of one individual were grouped with the putative parental lineages. For example, clones of the possibly hybrid "*A*. ×. *pseudo-heldreichii*" (individual **hd 1**) were grouped either with clones of *A. pseudoplatanus *(group A2) or of group A1 (*A. heldreichii, A. trautvetteri *and *A. velutinum*). Potential hybrid individuals (**hd 1**, **of 2**, **sv4b**) are directly identified in the splits graphs inferred from PBC distances, as they are placed between their putative parents; a large box-like structure with two prominent edge bundles is produced (Fig. 9).

"Recombinant" clones that are likely to be the product of PCR artefacts in the case of individuals with (potentially) homoeologous rDNA arrays [25] can distort phylogenetic trees and can induce a systematic attraction between distantly related lineages. In the case of *Acer*, four individuals exhibited chimeric clones, three of which are included in the present dataset (cf. [9]: Appendix B). From two *A. monspessulanum *specimens (**xx 3**, **xx 5**), recombinants between *A. monspessulanum*-and *A. opalus*-typical ITS variants were obtained; in the case of the *A. sempervirens *individual **sv4b**, a recombinant of *A. sempervirens *and *A. ibericum *("short variant") homoeologues was obtained. The recombinant clones obtained from *Acer *individuals do not have a distorting effect: In the case of **xx 3 **and **xx 5**, the recombinant clones are outnumbered and compensated for by *A. monspessulanum*-specific clones; in the case of **sv4b**, the recombinant perfectly fits into the general homoeologous situation.

Intra-individual ITS variability in *Fagus *is often as high as inter-individual or interspecific ITS divergence. Accordingly, the resolution of the phylogenetic trees was found to be low [24]; only detailed visual investigation of selected sequence motifs and ITS variability patterns [30,31] allowed us to infer a fully comprehensive evolutionary scenario in correlation with morphology and the fossil record [23]. The resultant PBC splits graph highlights several aspects of this data structure. As in phylogenetic trees [22,24,30], *F. engleriana *clusters with *F. japonica*, and individuals of *F. grandifolia *are clearly distinct from the remaining representatives of subgenus *Fagus*. In contrast to the ML phylogram based on the original clones [23], the strongly ITS-polymorphic individuals of *F. hayatae *and *F. longipetiolata *are grouped together (except for **lo 1 **and **lo 2**), which is in agreement with the results of the variability coding [31]. Individual **lo 1 **is placed next to *F. lucida*, which is in agreement with [23], who has noted that one *F. lucida *(not included in Fig. 10) has ITS characteristics that are more similar to *F. crenata *and *F. sylvatica*, whereas the other individual's (**lu 1**) ITS sequences have some similarities to some clones of *F. hayatae *and *F. longipetiolata*. Due to the increased intra- and inter-individual ITS variability in some provenances of the Western Eurasian *F. sylvatica *such as Turkey and Georgia [24], it was difficult to distinguish it clearly from its Japanese sister species, *F. crenata *[23,31]. This is, however, accomplished by the PBC-inferred distance networks (Fig. 10).

Ancient hybridization was assumed for the mostly diploid genus *Zelkova *[22]; however, the corresponding box-like structures are not readily visible in the PBC distance-based splits graph, because of the large box-like structure in the center of the graph (the giraffe's "torso" in Fig. 11). This is due to the fact that inter-taxonomic distances among clones of different *Zelkova *species are generally lower than, for instance, in *Acer*. In the former, apparently only a few mutations were fixed during the evolution of the modern species; accordingly, the Hamming distances between the clones are comparably small, as are the resultant PBC distances between the individuals. Nevertheless, the grouping of the "hybrid" and "non-hybrid" individuals is in perfect agreement with the proposed molecular evolution of *Zelkova *ITS [22]. The *Z*. cf. *serrata *cultivar **se 2**, genetically a hybrid of *Z. schneideriana *and *Z. serrata*, is placed in between *Z. schneideriana *individuals and the "true" *Z. serrata *representative, **se 5**. The second potential hybrid (**cp 1**) is represented by only two sequences in the original data and, hence, is not included in Figure 4. The "foreleg" position (Fig. 11) of the second *Z. carpinifolia *individual (**cp 2**) correlates to the finding based on motif analysis that the ITS of *Z. carpinifolia *is most similar to the ancestor of all *Zelkova *species [22].

## Conclusion

Four of the six formulae introduced in our study performed well in reconstructing the evolution of three angiosperm genera from cloned ITS sequences and of *Rosa *from three different coding loci. In conjunction with Delta values, the formulae were also able to identify hybrids and ancestral taxa in the ITS datasets. The four best-performing transformation functions apparently allow us to analyse datasets that present intrinsic problems for traditional reconstruction methods due to "paralogy" in molecular data, which range from ITS variability as the result of incomplete concerted evolution and homoeologous data reflecting reticulation, to gene paralogs as a consequence of gene duplication, not to mention homoploid duplications, incomplete lineage sorting, fast ancient radiations, etc.

A pure distance-based framework was established in which these transformation functions were assessed using three different approaches (Fig. 1), and the results regarding performance were in agreement with each other and also corresponded to the theoretical expectations. Furthermore, results obtained with the three *Rosa *sequence alignments were fully in agreement with an earlier study [33] that used a less general transformation function. Therefore, the outcome of the current study presents more than anecdotal evidence, even though only six underlying sequence datasets were examined. Nevertheless, additional investigations are necessary to further our understanding of these and other host-associate transformation functions. For instance, simulation studies could clarify the relative performance of the methods under well-defined evolutionary scenarios. In any case, our study has demonstrated that treelikeness measures such as Delta values are likely to be a valuable tool in future comparative studies, not only regarding entire distance matrices but also regarding the recovery of individual treelikeness values after transformation to distances between the hosts.

Furthermore, due to the generality demonstrated for the transformation functions, they can be applied to a wide range of biological problems that can be interpreted in terms of hosts and associates. For instance, Göker *et al*. are currently conducting a simulation study (which is not based on an explicit model of parasite evolution, but uses the approach of [56]) to assess the suitability of the PBC function for testing global co-phylogenetic patterns between hosts and parasites. Because of its closeness to the Sørensen coefficient, the PBC approach lends itself to use in ecology. In contrast to Sørensen distances, PBC does not require knowledge of the taxonomic affiliation of the sampled species in advance. Rather, it determines the most similar specimens between two habitats automatically when computing pair-wise distances from the reduced matrices (see below). PBC may thus be ideal for cluster analyses of habitats from which large samples of character data have been collected in a standardized manner, for instance, in the course of metagenomics projects (e.g. [65] and references therein). MOD may be helpful in phylogenetic studies to limit the number of OTUs by replacing numerous associates (members of a predefined taxon of a higher rank) with a single host (the respective taxon), probably losing much less information than if consensus or placeholder strategies were used. Various applications are possible, and further transformation formulae are likely to be invented in future studies benefiting from the general notion of associates related to their hosts.

## Methods

### Molecular character data – cloned ITS sequence matrices

Our analyses make use of the ITS data matrices by Grimm *et al*. for *Acer *section *Acer *[9], Denk et al. for *Fagus *[23] and Denk and Grimm for *Zelkova *[22]. The assembled ITS data cover genetic variability within the three model genera at different levels (intra-individual to inter-specific) and were obtained mainly from individuals growing in original stands. All individuals have been carefully revised taxonomically and form the basis for the morphological data matrices (in addition to individuals that were not sequenced). All three ITS matrices contain data complicating phylogenetic tree-building, such as chimeric sequences (potentially PCR-mediated recombinants in *Acer*), significant intra-individual variability (incomplete concerted evolution among ITS arrays in *Acer*, *Fagus *and *Zelkova*), and ITS homoeologues (genetic hybrids in *Acer *and *Zelkova*); the matrices comprise 242 (*Acer*), 137 (*Fagus*), or 55 (*Zelkova*) cloned sequences from 73 (*Acer*), 43 (*Fagus*), or 18 (*Zelkova*) individuals. That is, the number of clones per individual ranges from 1 to 10 (*Acer*) or 1 to 5 (*Fagus*, *Zelkova*). The gaps present in these matrices are relatively short (each gap is extended over only few characters); hence, treating each single gap symbol as a 5^th ^character state (see below) is unlikely to give too much weight to gaps. ITS alignments in NEXUS format are included in Additional file 1; for accession numbers, clone labeling and additional voucher information, refer to the original literature [9,22,23].

### Morphological character data – independent set of data matrices

For *Acer *section *Acer*, morphological characters related to leaf anatomy and morphology of reproductive organs (Table 2 in [9]) were assembled to discuss and interpret the results of the molecular analyses in a spatio-temporal framework, including evidence from the fossil record. This information has been transformed into a matrix of 15 characters and 13 OTUs ("species"; Additional file 5) using "STANDARD" characters [32] and differentiating up to seven character states (T. Denk, GWG, person. comm. 2007). For *Fagus *and *Zelkova*, we relied on the published data matrices of Denk ([59]: Table 4; 42 characters including features of leaf anatomy and morphology, cupule morphology and pollen characteristics. See also [23]) and Denk and Grimm ([22]: Table 4; 24 characters from leaf morphology, leaf anatomy, wood structure and flower and fruit characteristics). Characters of all three matrices are defined to address the discrimination or overlap between formerly recognized taxonomic entities ("species", "subspecies"); overlapping morphological features are represented by polymorphic character states. Morphological matrices in NEXUS format are included in Additional file 2, for details of the characters, refer to the original literature [22,23,59].

### Character and distance transformations

To conduct and assess the transformations examined, four computer programs were written by MG: G2CEF, which converts associate to host characters; PBC, which converts associate to host distances; EUKDIS, which calculates Euclidean distances; and DIST_STATS, which calculates Delta values. These programs have been implemented in the Ada 2005 language, have been compiled for the three main operating systems, and are freely available for download at [66]. The program versions used in the present study are also included in Additional Data File 6. The programs are able to read data exported from PHYLIP [67] or from PAUP* [32], which is currently the most popular phylogeny software, and (apart from DIST_STATS) are able to write data in formats readable by PAUP*, PHYLIP and SplitsTree [44] (in the case of discrete character or distance data) as well as by PhyML [68] and RAxML [69] (in the case of nucleotide character data). The programs can read from standard input and write to standard output, and can be used as stand-alone executables or integrated in larger analysis pipelines. For instance, in the course of our empirical assessments, they were called successively by a UNIX shell script, which is less portable but is available from MG upon request.

There are basically two ways to derive a host distance measure from the character data of the associates (Fig. 1). The first approach consists of transforming the associates' character matrix into a character matrix of the hosts and then inferring a distance matrix from these characters. In the following, we will describe four distance measures which follow that approach and can be applied if, for example, the associates' characters represent aligned sequence data. An alternative family of methods computes pair-wise host distances directly from an associate's distance matrix. The latter may represent phenetic distances derived from character data, or patristic (path-length) distances [55] derived from an associate tree or even from a list of taxonomic affiliations of the associates [70]. Below, we also introduce two host distance measures of the kind which can generally be applied.

**Figure 13:**
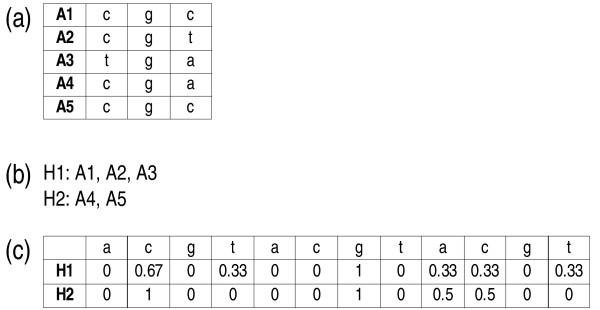
**FRQ character transformation: a numerical example**. This example illustrates the transformation of characters according to FRQ: (a) hypothetical three-character nucleotide alignment for the associates A1 to A5; (b) distribution of the associates on the hosts H1 and H2; (c) the three associate characters transformed to twelve (three times the number of character states) characters of the host. The standardized Euclidean distance between H1 and H2 calculated from these characters is 0.180.

A frequency (FRQ) character matrix is generated from aligned sequences (or any other matrix of discrete characters) of the associate by converting the parts of each alignment (matrix) column that correspond to the identical host into a vector representing the frequencies of each permitted character state (Fig. 13). Since unequal sampling of the associates may frequently occur in empirical studies, relative frequencies have to be used. The size of the resulting character matrix is the number of hosts times the number of characters in the original associate matrix times the number of valid character states (Fig. 13). Host-host distances may then be computed from such a matrix of quantitative, continuous characters using standard formulae, among which, average Euclidean distances (e.g., [60]: p. 278) as implemented in EUKDIS probably represent the simplest and most natural approach. As in the case of the transformations described below, states representing missing data are discarded by G2CEF before computing consensus sequences; the option -g regulates whether gaps are treated as missing data.

Entropy (ENT) distances are based on a character matrix representing the Shannon entropy [71] within each part of an alignment column that belongs to the same host. If q denotes the total number of character states and p_i _denotes the relative frequency of character state i, the Shannon entropy H (e.g., [50]: p. 240) is defined as

(1) $$H=-\sum_{i=1}^{q}p_{i}\log p_{i}.$$

The size of an ENT character matrix is the number of hosts times the number of characters in the original associate matrix. As in the case of FRQ, variance in the sampling size has to be accounted for. Thus, each entropy value is scaled by dividing by the exact maximum possible entropy H_max _for the given number of associates and valid character states. H_max _can be computed with ease by generating an artificial partial dataset in which the character states are distributed as equally as possible among the set of associates. This implies that ENT is undefined if fewer then two associates have been sampled from a host or else division by zero would occur. As in the case of FRQ, average Euclidean distances can be computed from such a matrix if characters with missing states are omitted from pair-wise comparisons and the total number of characters (the denominator of the distance function) is adjusted accordingly (-g option of the EUKDIS program). In contrast to FRQ, ENT discards all information about the original character state composition within each character and each host's associates, and relies solely on the observed variability within the respective character.

Consensus (CON) distances first apply ambiguity coding to all character states present in the set of a single host's associates and the respective character; in the case of DNA data, the standard DNA nucleotide ambiguity coding is applied. For example, if the individual's clones exhibit either a "C" (cytosine) or a "T" (thymine) at a certain position, the individual's consensus nucleotide is "Y" (for pyrimidine). Even though this kind of coding is confined to DNA data, corresponding state recoding is possible with each kind of qualitative data; for instance, in the NEXUS standard as used by PAUP* [32], "Y" is just a placeholder for "{CT}". Genetic distances based on consensus sequences can be calculated using the same variety of approaches as for original sequences, based on more or less complex statistical models of DNA evolution [54,61]. In the course of our analyses, we calculated simple uncorrected (Hamming) distances with PAUP* (DSET DIST = P) because in preliminary analyses (not shown), we observed that with the current data, the type of recoding has a much greater effect on the correlation with reference distances and on the Delta values than the nucleotide distance function applied. Furthermore, more complex models need not result in smaller Delta values [47,49] and the Mantel test for congruence (e.g., [60]: p. 552; see below) transforms distances to ranks, which would give identical results for at least a considerable part of nucleotide distance formulae. Uncorrected distances that make use of gap characters are not implemented in PAUP*. To examine the effect of considering gaps as a 5^th ^character state rather than as missing data, we therefore recoded the data to "STANDARD" characters ([32]; FORMAT DATATYPE = STANDARD), explicitly set the ambiguity code placeholders, and computed the mean distances (DSET DIST = MEAN).

Mode (MOD) distances first determine the most frequent value (i.e. the mode. See [60]: p. 185; [62]: p. 48) for each host's associates and each character. If ties occur, a consensus character state is calculated from all modes using the same algorithm as in the CON method (i.e., in the case of nucleotide data, standard DNA ambiguity coding is applied). CON, which uses all present character states; and MOD, which considers only the modes, thus represent the two extremes of constructing a consensus character state. Distances from MOD characters were computed as for the CON method.

Another approach to derive a host distance measure from character data of the associates is first to compute the distances between the associates, which are then transformed to distances between the hosts. For the reasons provided above and to increase the comparability between the following and the above-mentioned methods, we also considered uncorrected ("p") distances between the associates only.

To determine the distance D_xy _between two hosts x and y, the first step in each of the following two distance formulae is to omit all rows which correspond to non-associates of x from the associates' squared distance matrix and to omit all columns which correspond to non-associates of y from the matrix. Thus, a reduced matrix is obtained, the size of which is the number of associates of x times the number of associates of y. In the following formulae, let d_ij _be the distance between associates i and j, let A_x _be the complete set of associates of host x, and let A_y _be the complete set of those of host y. The distance functions now differ in how the distance between x and y is determined from the distance values within the reduced matrix. The MIN function computes their minimum:

(2) *D*_*xy*_: = *min*(*d*_*ij*_|*i *∈ *A*_*x*_, *j *∈ *A*_*y*_)

If two hosts are characterized by exactly the same set of associates, the minimum values are the diagonal zeros of the associate distance matrix. This ensures that D_xy _is always zero if x and y are identical, a necessary prerequisite for considering a formula a distance function (e.g., [60]: p. 274). In contrast, formulae based on the arithmetic mean, the median or the maximum of the distance values contained in the reduced matrix do not have this property. On the other hand, the MIN distance can be zero between two hosts with *different *sets of associates because a single shared associate will result in a zero value present in the reduced matrix. Because the distance function described in [33] uses averaging, MIN results are different in the case of two associates per host, unless the distances to the two distinct associates are identical. The more elaborate PBC ("phylogenetic Bray-Curtis") approach determines the mean of all row and column minima, thus considering, for each associate of host x, the most closely related (least distant) associate of host y only, and vice versa:

(3) $$D_{xy}:=\left(\sum_{i\in A_{x}}min\left(d_{ij}|j\in A_{y}\right)+\sum_{i\in A_{y}}min\left(d_{ij}|j\in A_{x}\right)\right)/\left(\left|A_{x}|+|A_{y}\right|\right).$$

**Figure 14:**
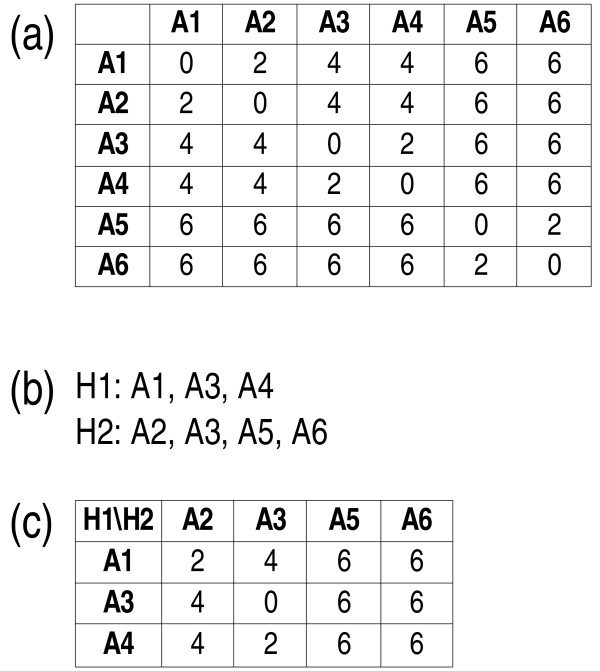
**Computation of PBC distances: a numerical example**. This example illustrates the computation of PBC distances: (a) distances between the associates A1 to A6; (b) distribution of the associates on the hosts H1 and H2; (c) reduced matrix including in the rows associates of host H1 only and associates of host H2 only in the columns. The row minima are 2, 0, and 2; the column minima are 2, 0, 6, and 6. The PBC distance between H1 and H2 is (2+0+2+2+0+6+6)/(3+4) = 18/7 = 2.571.

The symbol |A_x_| denotes the number of members in A_x_. A numerical example for PBC is presented in Fig. 14. According to Formula 3, D_xy _is zero if and only if A_x _and A_y _are identical because otherwise at least one of the row or column minima, which have to be considered in the computation of the average, would be larger than zero. Other properties of the PBC function, as well as the origin of its name, are explained in the discussion section. The PBC, applied to hosts with either one or two associates, is not identical to the distance function described in [33] (which cannot readily be generalized to more than two associates per host) because the associate distance values to be averaged are selected in a different manner.

### Distance quality estimation, correlation with external data and detection of sampling size bias

The resultant distance matrices would allow us to infer phylogenetic trees, e.g. by using the Neighbor-joining algorithm [72]. However, for the reasons given in the background section, we relied on the Neighbor-Net algorithm [73,74] as implemented in SplitsTree version 4.8 [44] to compute splits graphs based on the resultant distance matrices.

Delta values [47-49] for the complete matrices as well as individual OTUs (-i option) were computed with the program DIST_STATS. The DIST_STATS executables are expected to give the same results for Delta values as the Python script used in [48], but are expected to run much faster (approximately by a factor of 30) because they are written in a compiled language and the distance matrix is stored in a linear array. Delta values are based on quartets of taxa, and range between 0 (maximum treelikeness) and 1. Whereas Delta values (DV) are calculated by averaging among all quartets in a dataset, individual Delta values (iDV) are obtained by averaging among all quartets that include the respective OTUs. There is exactly one DV per distance matrix, but there are as many iDV as OTUs per distance matrix; for further details, see [48] and [49].

For the correlation of molecular distance data with morphology, uncorrected distances were calculated with PAUP* from the morphological matrices described above. The congruence between distance matrices was assessed using the CADM software [52]. Ordinary significance tests cannot be applied because entries in a distance matrix do not represent statistically independent characters. Hence, CADM uses permutation (Mantel test; e.g. [60], p. 552) to determine the significance of the Spearman's rank correlation between the distance matrices [51]; we used 999 permutations of the original matrices. (The software also implements a test for the congruence of several matrices, which was not applied.)

To detect a potential bias related to the sampling size, i.e. the number of associates (clones obtained) per host (plant individual), G2CEF and EUKDIS were used to calculate Euclidean distances between the numbers of associates per host. The resulting SIZ distance matrices were included in the above-mentioned CADM permutation runs, the rationale being that if any of the other distance functions significantly correlated with the sample-size distances, the outcome needs to be considered as biased. Based on preliminary observations, the complete set of computations was conducted for minimum group size values of 2, 3 and 4; hosts with a lower number of associates in the dataset were automatically removed prior to analysis. These group-size thresholds can be applied automatically in both PBC and G2CEF (-m option). We determined the smallest threshold, for which a correlation of SIZ significant at p = 0.05 with any of the other transformed distance matrices was not observed. Distances matrices obtained with group sizes greater than this threshold value were used for all visual comparisons of networks and all boxplots.

As an additional empirical assessment, and to compare our results to those obtained by Joly and Bruneau [33], we applied the six transformations to the three datasets examined therein, which were downloaded from [75]. The data contain glyceraldehyde 3-phosphate dehydrogenase (matrix M2598; 66 sequences), triose phosphate isomerase (matrix M2600; 69 sequences) and malate synthase (matrix M2599; 66 sequences) alleles obtained from North American *Rosa *(Rosales, Rosaceae) individuals. Since external independent datasets are unavailable for these specimens, we treated them separately, confining ourselves to calculating Delta values and visually comparing their networks to those presented in [33]. In contrast to the *Acer*, *Fagus *and *Zelkova *datasets (in which the number of associates per host is determined by the experimental success in PCR, cloning and sequencing) in the *Rosa *datasets, the number of associates per host represents the alleged number of alleles (one or two) per individual. Therefore, for the *Rosa *data, the minimum number of associates required was set to two and the sequences of the single-allele host were duplicated. (This is necessary for ENT only; the other transformations would give identical results without duplication and with a threshold of one.) Furthermore, because results are identical between CON and MOD when there were one or two associates per hosts, CON distances were not calculated.

## Abbreviations

OTU, operational taxonomic unit; DV, Delta value of an entire distance matrix; iDV, Delta value of a single OTU; CON, character transformation using consensus sequences; ENT, character transformation to entropy values; FRQ, character transformation to character state frequencies; MIN, distance transformation using minimum distances between associates; MOD, character transformation to modes of character states; PBC, "phylogenetic Bray-Curtis" distance transformation; SIZ, distances derived from associate numbers.

## Authors' contributions

MG designed, implemented and evaluated the character and distance transformation functions; implemented the computation of Delta values and Euclidean distances; and designed the pure distance-based testing framework. GWG provided the data and expertise on the focus genera. MG and GWG interpreted the results, and drafted and wrote the manuscript. All authors read and approved the final manuscript.

## Supplementary Material

Additional file 1 **Distance matrices and quality statistics**. This contains all sequence alignments in NEXUS format, all intermediary generated associate distance or host character data (depending on the transformation formula used; see Fig. 1) and all final distance matrices obtained, together with their Delta values. A readme file that describes the contents of each file in the archive is included. Click here for file

Additional file 2 **Correlation with morphology**. This contains the morphological character data in NEXUS format for the three genera examined, matrices of uncorrected ("p") distances computed from these characters, and the correlation results and permutational probabilities obtained by applying CADM to these distance files and molecular distances (which are contained in supplementary data file 1). A readme file that describes the files contained in the archive is included. Click here for file

Additional file 3 **Distance matrices and quality statistics obtained with the *Rosa *dataset of Joly and Bruneau **[33]. This contains all intermediary generated associate distance or host character data (depending on the transformation formula used; see Fig. 1) and all final distance matrices obtained together with their Delta values. A readme file that describes the contents of each file in the archive is included. The corresponding NEXUS files containing the original associate sequence data (alleles of North American *Rosa *species) are available at [75] as study No. S1444. Click here for file

Additional file 4 **Individual Delta values**. This lists the iDV inferred from FRQ, MIN, MOD and PBC distances using a threshold of 3 and treating gaps as missing character states, as well as iDV inferred from p distances between the cloned sequences, also treating gaps as missing character states. A readme file that describes the files contained in the archive is included. Click here for file

Additional file 5 **Morphological characters coded for *Acer *section *Acer***. Morphological characters listed for *Acer *section *Acer *in [9], Table 2, and coding used for this study. Click here for file

Additional file 6 **Main programs implemented in the course of the study**. Contains executables for UNIX/Linux, Mac OS-X and Windows for the programs G2CEF, PBC, EUKDIS and DIST_STATS, as well as example input files corresponding to the different data formats. Click here for file
